# The Retrobulbar Spot Sign and Prominent Middle Limiting Membrane as Prognostic Markers in Non-Arteritic Retinal Artery Occlusion

**DOI:** 10.3390/jcm10020338

**Published:** 2021-01-18

**Authors:** Marlena Schnieder, Charlotte V. Fischer-Wedi, Sebastian Bemme, Mai-Linh Kortleben, Nicolas Feltgen, Jan Liman

**Affiliations:** 1 Department of Neurology, University Medical Center Göttingen, 37073 Göttingen, Germany; 2 Department of Ophthalmology, University Medical Center Göttingen, 37073 Göttingen, Germany; c.fischer@med.uni-goettingen.de (C.V.F.-W.); sebastian.bemme@med.uni-goettingen.de (S.B.); mai-linh.kortleben@med.uni-goettingen.de (M.-L.K.); nicolas.feltgen@med.uni-goettingen.de (N.F.)

**Keywords:** sudden vision loss, imaging, optical coherence tomography, prognosis, visual acuity, central retinal artery occlusion

## Abstract

Central retinal artery occlusion (CRAO) is characterized by the sudden, painless loss of vision. Typical sonographic and optic coherence tomography (OCT) findings are a retrobulbar spot sign and prominent middle limiting membrane (p-MLM) sign. It remains uncertain whether the retrobulbar spot sign alone or coinciding with the appearance of p-MLM sign is a prognostic marker for visual acuity and the development of secondary retinal ischemia after CRAO. In our prospective cohort study, we included patients with a non-arteritic central artery occlusion < 4 weeks. We examined the following parameters at prespecified time points: ultrasound examination of orbital cavity, Spectral Domain-OCT examination, visual acuity test, and fundoscopy and ultra-widefield angiography to diagnose retinal vascularization. The presence of p-MLM sign in SD-OCT after CRAO was accompanied by significantly better vision during the first four weeks (2.3 (IQR 0.75) vs. 2.6 (IQR 0.33); *p* = 0.006). Moreover, the spot sign seems to be a prognostic factor for developing secondary retinal ischemia (8 (100%) vs. 0 (0%); *p* = 0.036). A retrobulbar spot sign seems to be a negative prognostic factor and is associated with secondary retinal ischemia, whereas a p-MLM sign is a somewhat positive prognostic factor for visual acuity.

## 1. Introduction

Central retinal artery occlusion (CRAO) is characterized by sudden, painless monocular vision loss [1]. The etiologies of CRAO are classified as arteritic or non-arteritic, with the latter caused by an embolus [2]. Patients suffering from non-arteritic CRAO share risk factors resembling those in patients suffering heart attack or stroke [3], and an increased cardiovascular risk has been diagnosed in 78% during post-CRAO clinical workup. Atrial fibrillation is diagnosed in both patients with CRAO and those suffering ischemic stroke [4], although it seems to be less frequent in the latter group. CRAO patients are more likely to have valvular disease and smoke than ischemic stroke patients [5]. Patients with CRAO do not just share the same cardiovascular risk factors as ischemic stroke patients: their stroke risk is 2.7-fold higher than the normal population’s [6]. Stroke or transient ischemic attack often occurs soon before or after a CRAO [7]. A meta-analysis revealed that 30% of patients with CRAO also suffered from acute cerebral ischemia in magnet resonance imaging (MRI) [8]. A retrospective analysis showed that up to 19.5% of patients suffering monocular vision loss of vascular etiology had also had a silent brain infarction in MRI [9]. The prognosis for visual acuity following CRAO is usually poor [2]. There are few current treatment options after CRAO, and none has proven capable of alleviating visual loss significantly [10]. Therapy options for acute CRAO are ocular massage, anterior chamber paracentesis, carbogen therapy, or intravenous treatment with acetazolamide or mannitol, as well as more aggressive approaches such a thrombolysis or Nd:YAG laser embolectomy [11,12]. Thrombolysis with recombinant tissue plasminogen activator (rtPA) in particular has delivered inconsistent results [13,14,15]. A retrobulbar spot sign in retro-orbital sonography restricted exclusively to non-arteritic CRAO is considered the correlate of a calcified embolus [16]. Consistent with this, patients presenting a retrobulbar spot sign fail to benefit from systemic thrombolysis [17]. A point-of-care ultrasound of the orbital cavity enabling screening for a spot sign is a useful addition when diagnosing CRAO [18], and it may help to determine the CRAO’s etiology and even point out those patients more likely to show a positive rtPA response [19]. There is evidence that a prominent middle limiting membrane (p-MLM) sign is an indicator for acute ischemic change in spectral domain optical coherence tomography (SD-OCT) [20]. Yet we still do not know whether the retrobulbar spot sign alone or in combination with the appearance of p-MLM sign is a prognostic marker for visual acuity and the development of secondary retinal ischemia after CRAO.

## 2. Materials and Methods

This study had a prospective arm to analyze the prognostic value of both the spot and p-MLM signs. We also carried out a retrospective analysis to identify any coincidence between the spot sign and cerebrovascular vascular risk factors in a larger cohort.

### Prospective Trial

From December 2015 until April 2019, we enrolled all suitable patients with a recanalized or complete CRAO in this prospective, longitudinal study.

#### Inclusion and Exclusion Criteria

Inclusion criteria were a non-arteritic central or hemi-central retinal artery occlusion with first symptoms within the last four weeks. Patients had to be 18 years old or older.

Exclusion criteria were optic disc drusen and diagnosis of an arteritic CRAO as well as a cilioretinal artery supplying the macula.

The study was approved by our local ethics committee (study number 13/9/15); informed consent was obtained from all patients. Patients were followed-up four and 12 weeks after inclusion. At the timepoint of inclusion, all patients underwent a thorough ophthalmological and ultrasound examination of the orbital cavity. Ultrasounds were done on a GE Logiq S8 (General Electrics, Boston, MA, USA) in B-mode with a 9 mm linear transducer. The acoustic output of the mechanical index was as low as possible. Best-corrected visual acuity (BCVA) was assessed using the logMAR scale. Low visual acuity as “hands count” and “light perception” were converted into logMAR as previously published [21,22]. The anterior segment was examined by slit lamp and eye pressure, and we assessed any relative afferent pupillary defect. Every patient underwent fundoscopy, SD-OCT and ultra-widefield retinal fluorescein angiography (UWFA). SD-OCT examination (Spectralis, Heidelberg Engineering, Heidelberg, Germany) covered the macula by 25 B-scan-lines (6 mm) with every scan line 200 µm apart. P-MLM sign was evaluated in all 25 SD-OCT scans. For evaluation the scan-quality had to be above 20 points and p-MLM sign had to be found in at least 50% of all scans within a radius of 1000 µm from the foveal center. UWFA was performed using a 102°-wide field imaging camera (Heidelberg Engineering, Heidelberg, Germany) to detect secondary ischemia visible as capillary non-perfusion.

BCVA testing, fundoscopy and SD-OCT examination were done at inclusion and again four and 12 weeks later. UWFA was performed at inclusion and repeated after 12 weeks. We also collected baseline characteristics and information on the etiology of the non-arteritic CRAOs. We relied on the TOAST-Criteria [23] to define CRAO etiology. All examinations were performed by experienced neurologists (ultrasound) and ophthalmologists.

#### Retrospective Trial

Apart from this prospective trial, we also conducted a retrospective analysis of the etiology of CRAO in patients with a spot sign to enlarge the patient group. This was possible because all of our CRAO patients since 2015 have undergone ultrasound to check for the spot sign. We collected the data on patients with a CRAO and their ultrasound orbital-cavity examinations from 2015 until June 2020, and analyzed these to determine their CRAOs’ etiologies in conjunction with the absence or presence of a spot sign.

Descriptive statistics are presented using mean and standard deviation or median and interquartile range as adequate. Groups comparisons between categorical variables were made using chi-squared or Fischer’s exact test, respectively. To compare ordinally scaled data such as BCVA, we applied the Mann–Whitney *U*-test. Results with a *p*-value < 0.05 were considered statistically significant. Statistical analysis was conducted using IBM SPSS Statistics vs 26 (IBM US, Armonk, New York, NY, USA).

## 3. Results

### 3.1. Prospective Trial

**Figure 1:**
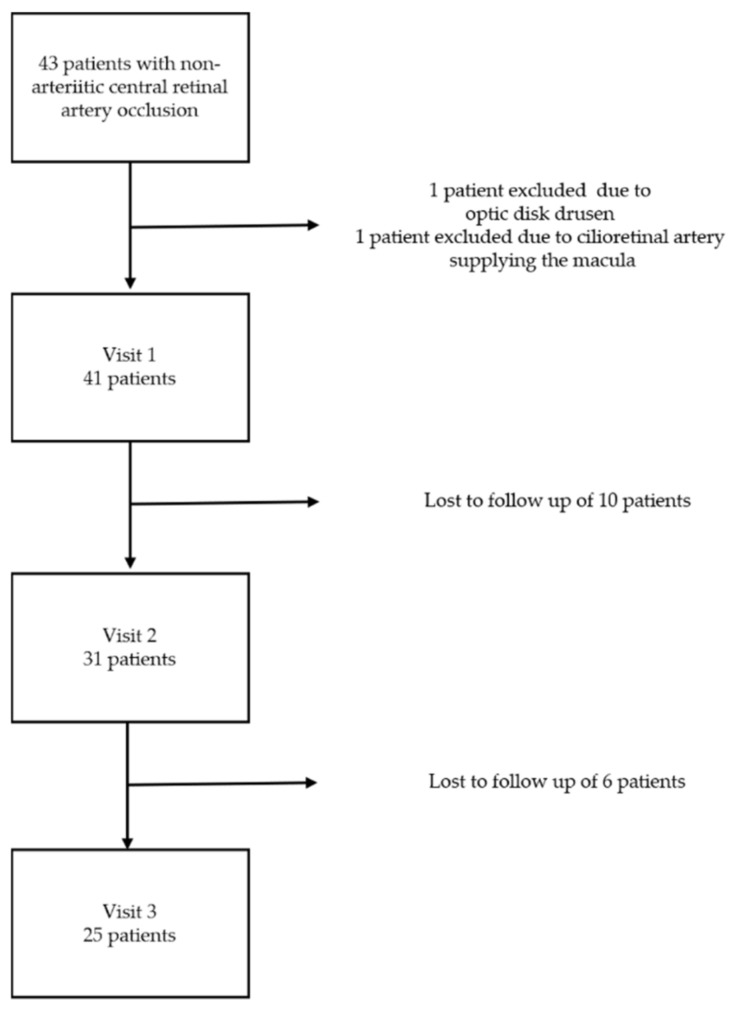
Flow-diagram of the study design.

From December 2015 until January 2019, we prospectively included 43 patients in all with non-arteritic CRAO in this study. Due to those lost in follow-up, there is incomplete data on 17 patients, and one patient had to be excluded due to optic disc drusen and one other patient had to be excluded due to a cilioretinal artery supplying the macula. A total of 25 patients completed all visits (Figure 1).

**Figure 2:**
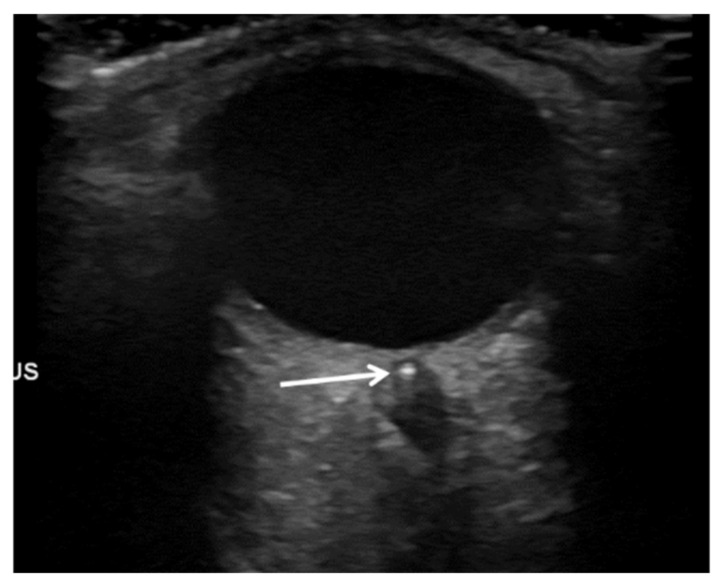
Ultrasound of orbital cavity with a retrobulbar spot sign (arrow) in central retinal artery.

Twenty-seven (65.9%) patients were male and 14 (34.1%) female. The left eye was affected in 18 (43.9%) patients, and in 23 (56.1%) the right eye. A spot sign was diagnosed in 24 (58.5%) patients (Figure 2).

**Figure 3:**
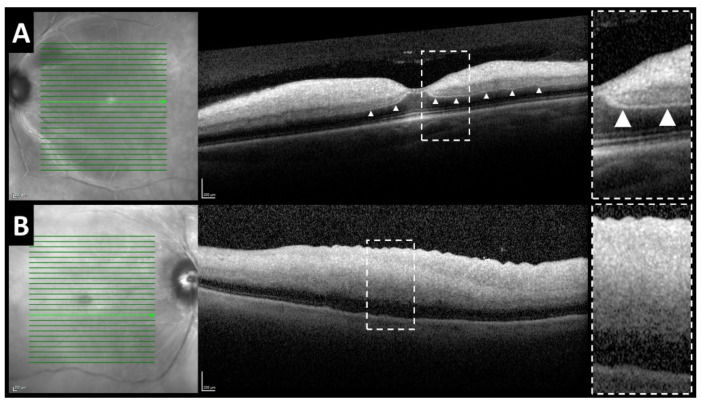
SD-OCT cross-sections after central retinal artery occlusion with visible p-MLM sign ((**A**) arrowheads) and with maximum increase in reflectivity of the entire inner retina inhibiting identification of p-MLM sign (**B**).

A p-MLM sign was visible on SD-OCT in 26 patients (63.4%) (Figure 3A), in 11 patients (26.8%) p-MLM could not be identified due to increased reflectivity of the entire inner retina (Figure 3B) (in four patients OCT was missing).

Of our included patients, 13 (31.7%) showed a recanalized CRAO and 25 (65.7%) had a persistent artery occlusion (in three patients recanalization or persistent occlusion could not be determined). Eight (44%) presented retinal capillary ischemia in UWFA. Two patients had a perfused cilioretinal artery one of those affecting the macula, which led to the subsequent exclusion of that patient. CRAO etiology classified according to the TOAST-Criteria revealed that 10 (24.4%) were macroangiopathic with a >50% ipsilateral carotid stenosis in line with the North American Symptomatic Carotid Endarterectomy Trial (NASCET) [24], six (14.6%) were cardio-embolic, five (12.2%) microangiopathic, two (4.9%) revealed another etiology; no etiology was identified in 18 patients (43.9%) whereby the etiology of CRAO remained cryptogenic (Table 1).

**Figure 4:**
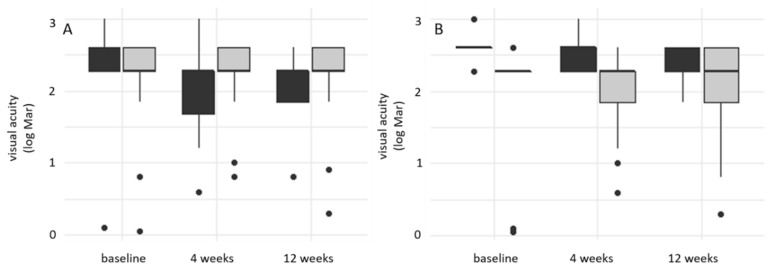
(**A**) box plot of BCVA (log Mar) in patients with p-MLM sign (gray) and without (dark) (visit 1: *p* = 0.0012; visit 2: *p* = 0.001; visit 3: *p* = 0.392); (**B**) box plot of visual acuity (log Mar) in patients with spot sign (gray) and without (dark) (visit 1: *p* = 0.548; visit 2: *p* = 0.357; visit 3: *p* = 0.190).

At the first visit and inclusion timepoint, median BCVA of all patients was 2.27 (IQR 1.28); the BCVA of patients presenting a p-MLM sign was significantly better than the BCVA of patients without (2.3 (IQR 0.17) vs. 2.6 (IQR 0.00); *p* = 0.012) (Figure 4A). The same held true at visit 2 after four weeks (2.3 (IQR 0.75) vs. 2.6 (IQR 0.33); *p* =0.001). After 90 days, we found no difference in BCVA (2.3 (IQR 0.76) vs. 2.4 (IQR 0.33); *p* = 0.392) comparing patients with or without p-MLM. Regarding the retrobulbar spot sign (Figure 4B): BCVA did not differ at visit 1/inclusion timepoint (2.3 (IQR 0.33) vs. 2.4 (IQR .33); *p* = 0.548), at visit 2 (2.3 (IQR 0.33) vs. 2.3 (IQR 0.91); *p* = 0.357) or at visit 3 after 90 days (2.3 (IQR 0.33) vs. 2.3 (IQR 0.43); *p* = 0.190) in patients presenting a spot sign compared to those without one.

**Figure 5:**
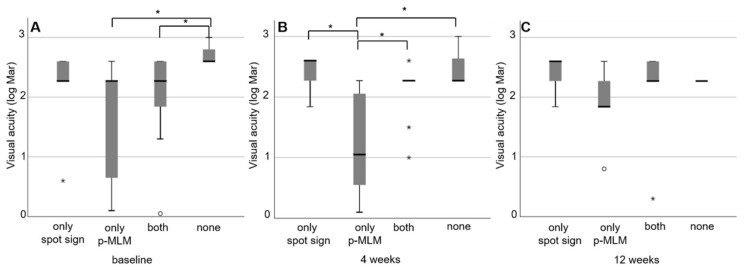
Box plot of visual acuity grouped by different factors (spot sign, p-MLM sign, both and none) at (**A**) visit 1; (**B**) visit 2 and (**C**) visit 3; * *p* < 0.05.

Our analysis of both the p-MLM sign and spot sign combined showed significantly better visual acuity at visit 1 in patients revealing both factors compared to those without either factor (2.3 (IQR 0.89) vs. 2.6 (IQR 0); *p* = 0.031); it also reveals significantly better visual acuity in patients with a p-MLM sign and no spot sign than in those without either factor (2.3 (IQR 1.25) vs. 2.6 (IQR 0); *p* = 0.006). At visit 2, a p-MLM sign without a spot sign was always significantly better than a p-MLM sign and spot sign (1.84 (IQR 1.37) vs. 2.3 (0.4); *p* = 0.044), no factor (1.84 (IQR 1.37) vs. 2.3 (IQR 0); *p* = 0.015) or spot sign alone (1.84 (IQR 1.37) vs. 2.6 (IQR 0.33); *p* = 0.001). We detected no significant difference in any of the groups at visit 3 (*p* = 0.329) (Figure 5).

**Figure 6:**
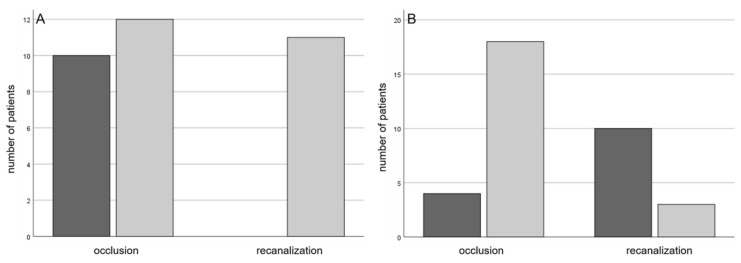
(**A**) number of patients with a p-MLM (grey) or without (dark) with a persistent occlusion or recanalization of the central retinal artery, *p* = 0.013; (**B**) number of patients with a spot sign (grey) or without (dark) with a persistent occlusion or recanalization of the central retinal artery, *p* = 0.001.

Considering the secondary complications of CRAO: two patients had neovascular complications within the follow-up period. One was treated with panretinal photocoagulation and one was operated due to vitreous hemorrhage. All patients suffering capillary nonperfusion confirmed by UWFA revealed a spot sign in the retrobulbar ultrasound (8 (100%) vs. 0 (0%); *p* = 0.036), whereas only four (50%) of the patients with secondary retinal ischemia presented a p-MLM sign. Moreover, we observed that the patients presenting a p-MLM sign tended to suffer from secondary retinal ischemia less often than those without one (4 (36.4%) vs. 7 (63.6%); *p* = 0.077). We detected no significant difference in the occurrence of secondary retinal ischemia in patients with a spot sign (4 (44.4%) vs. 5 (55.6%); *p* = 0.221). Eleven of 13 patients diagnosed with a spontaneously recanalized CRAO had a p-MLM sign (100%; in two patients OCT was missing), while patients with persistent CRAO showed a p-MLM sign in 14 of 25 cases (58.3%; *p* = 0.015; in one patient OCT was missing). The majority of patients suffering a persistently occluded central retinal artery had a spot sign (18 (81.8%); *p* = 0.001) (Figure 6).

**Figure 7:**
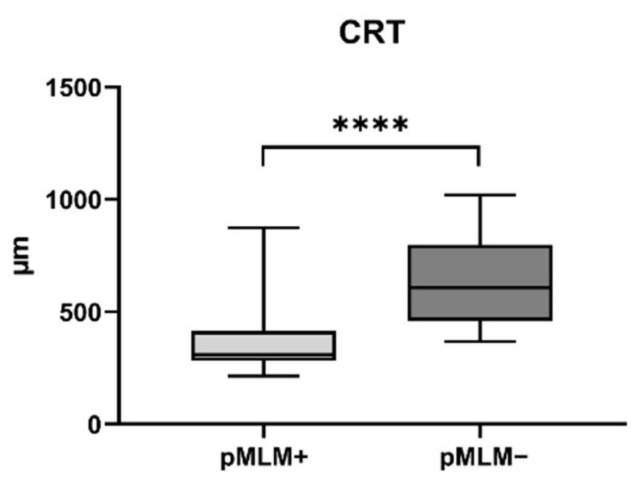
Box plot of central retinal thickness of patients with or without a p-MLM. **** = *p* < 0.001. CRT: central retinal thickness.

The central retinal thickness (mean ± SD, µm) was significant less in patients presenting with a p-MLM sign (357.4 ± 135.7 vs. 636.7 ± 201.7; *p* < 0.001) (Figure 7).

There was no significant difference in CRAO etiology in patients with a spot sign (*p* = 0.27) or a p-MLM (*p* = 0.37). Patients with a spot sign had a macroangiopathic CRAO etiology in six (30%) cases, cardio-embolic etiology in five (25%), micro-angiopathic etiology in two (10%), other etiology in two (10%) cases and in five (25%) cases the etiology remained cryptogenic. Whereas patients with a p-MLM had in seven (30.4%) cases a macroangiopathic etiology, in four (17.4%) cases a cardio-embolic etiology, in four (17.4%) a microangiopathic etiology, and another etiology was identified in two (8.3%) cases. The CRAO etiology remained cryptogenic in six (26.1%) patients.

### 3.2. Retrospective Trial

Our retrospective analysis of all patients with CRAO and an orbital-cavity ultrasound contained 106 patients. Of those 106 patients, 54 (50.9%) revealed a spot sign in the ultrasound examination. A macro-angiopathic etiology was determined in 29 (27.4%) patients, cardio-embolic in 22 (20.8%), micro-angiopathic in six (5.7%) and another defined etiology in seven (6.6%) patients. The CRAO remained cryptogenic in 42 (39.6%) with no specific etiology. Our comparison of patients with a spot sign to those without in etiological terms revealed no significant difference (macro-angiopathic (15 (28.8%) vs. 14 (25.9%)), cardio-embolic (7 (13.5%) vs. 15 (27.8%)), micro-angiopathic (4 (7.7%) vs. 2 (3.7%)), other defined etiology (3 (5.8%) vs. 4 (7.4%)), cryptogenic (23 (44.2%) vs. 19 (35.2%)); *p* = 0.413).

## 4. Discussion

We believe that the appearance of both spot and p-MLM signs could serve as a prognostic marker in patients suffering from an acute CRAO. The retrobulbar spot sign seems to be associated with secondary retinal ischemia, whereas the p-MLM sign seems to be an indicator for less severe ischemic damage. When both factors appear in combination, the early positive impact of the p-MLM sign remains apparent. The spot sign functioning as a negative prognostic factor is in line with other studies demonstrating that these patients reveal low endogenous recanalization rates [19] and do not benefit from systemical thrombolysis [17]. The poor visual-acuity outcomes and lack of response to intravenous thrombolysis might be attributable to the fact that the spot sign is the sonographic correlate of a calcified or cholesterol embolus [16]. We observed low spontaneous recanalization rates in the central retinal artery in patients with spot sign in our study as well, as well as a higher significant rate of secondary retinal ischemia in patients with a spot sign. The reason for this higher secondary ischemia rate is unknown. Low spontaneous recanalization rates and persistent spot signs in sonography [19] might help explain our study’s significantly higher rate of secondary ischemia. Unlike our patients with a spot sign, most patients with spontaneous recanalization showed a p-MLM sign. This may be evidence of the p-MLM sign’s function as a potential positive factor and as a marker for a less severe retinal ischemia. P-MLM was first described by Chu et al. in 2013 in 18 patients suffering retinal ischemic diseases [20]. They speculated that a p-MLM visible in SD-OCT represents acute swelling of predominantly bipolar cells synapses in the inner part of the retinal outer plexiform layer, resulting in a hyperreflective line in OCT compared to the less ischemic outer retinal layers, which are supplied by the choroid. The p-MLM sign is known to be associated with a poorer visual-acuity prognosis in conjunction with central retinal vein occlusion [25]. p-MLM sign serves as an indicator of severe central retinal vein occlusion with a retinal ischemia as a complication. Whereas in a severe retinal ischemia, all cells of the inner retinal layers suffer from acute swelling leading to a hyperreflectivity of the whole inner retinal layers in SD-OCT. As a result, p-MLM sign is no longer visible in SD-OCT due to the higher reflectivity of the other inner retinal layers. Therefore, in contrast to central retinal vein occlusion p-MLM sign is an indicator of less severe ischemic damage and might be a factor associated with a favorable outcome in visual acuity. This is reflected by the significant less central macular thickness in the group of patients with a p-MLM sign. Central macular thickness has been shown to be positively correlated with BCVA, with inverse correlation of macula swelling with visual acuity, the more severe the swelling, the visual acuity worsens [26]. However, there is data, were this correlation could not be shown [27]. A p-MLM sign can add useful information regarding this question.

CRAO is often associated with carotid stenosis. The distribution of CRAO etiologies in our study differs from those in normal stroke populations; our patients’ rate of carotid stenosis in particular was higher than a normal stroke population’s [28], but we detected no etiological difference in patients with p-MLM or spot sign. Patients presenting a CRAO with an ipsilateral carotid stenosis should undergo a complete clinical stroke workup, as competing CRAO etiologies are possible [29]. There is evidence that a spot sign can serve as a marker for an atherosclerotic embolus and CRAO etiology [18], but we detected patients presenting a CRAO of cardio-embolic origin and a spot sign as well. Nevertheless, the numbers of cardio-embolic CRAOs in patients with a spot sign are lower than in the normal stroke population [28]. Our study has several limitations: the main one being its small sample, due to the low number of patients suffering CRAO admitted to hospital. Another problem is the high number of patients lost to follow-up, which could have biased our visual-acuity findings.

## 5. Conclusions

A retrobulbar spot sign seems to be a negative prognostic factor associated with secondary retinal ischemia, whereas the p-MLM sign is correlated with a less severe ischemic damage.

## Figures and Tables

**Table 1:** Baseline characteristics of all patients.

Baseline Characteristics	
age (years; IQR)	75 (11)
female	14 (34.1%)
male	27 (65.9%)
left eye	18 (43.9%)
right eye	23 (56.1%)
spot sign	24 (58.5%)
p-MLM sign	26 (63.4%)
spot sign and p-MLM sign	11(26.8%)
secondary retinal ischemia confirmed by UWFA	8/18 (44%)
recanalization	13 (31.7%)
central macular thickness in µm (std)	507.8 (255.6)
central macular volume in mm^2^ (std)	0.4267 (0.19)
time since onset of symptoms (hours; IQR)	8 (17)
hypertension	27 (64.3%)
hyperlipidemia	20 (47.6%)
diabetes mellitus	7 (16.7%)
anti-coagulant medication	6 (14.3%)
**Etiology**	
macroangiopathy	10 (24.4%)
cardio-embolic	6 (14.6%)
microangiopathy	5 (12.2%)
other	2 (4.9%)
cryptogenic	18 (43.9%)

IQR = interquartile range, p-MLM = prominent middle limiting membrane, std: standard deviation.

## Data Availability

The data presented in this study are available on request from the corresponding author. The data are not publicly available to respect privacy of the patients.
